# Colorectal cancer severity and survival in correlation with tumour necrosis factor-alpha

**DOI:** 10.1080/13102818.2014.965047

**Published:** 2014-10-29

**Authors:** Noyko Stanilov, Lyuba Miteva, Zlatka Dobreva, Spaska Stanilova

**Affiliations:** ^a^ Department of Neurosurgery, Surgery and Urology, University Hospital, Trakia University, Stara Zagora, Bulgaria; ^b^ Department of Colorectal Surgery, Milton Keynes Hospital, Milton Keynes, UK; ^c^ Department of Molecular Biology, Immunology and Medical Genetics, Medical Faculty, Trakia University, Stara Zagora, Bulgaria

**Keywords:** cytokine production, tumour progression, inflammation, single nucleotide polymorphism

## Abstract

Colorectal cancer (CRC) development is strongly associated with innate immune mechanisms and intestinal inflammation. The aim of the study was to investigate the pre-operative serum levels of TNF-α and its correlation with cancer progression and survival in CRC patients taking into account the genotype of –308G/A promoter polymorphism in TNF-α gene (rs1800629). TNF-α –308G/A genotypes of 119 CRC cases and 177 no CRC controls were determined by restriction fragment length polymorphism assay (RFLP-PCR). TNF-α serum levels were measured by enzyme-linked immunosorbent assay (ELISA). Although no significant differences in allele and genotype frequencies between CRC and controls were observed, it should be noted that the minor allele-A and its homozygous genotype were overrepresented among CRC. In addition, allele-A was more frequent in early CRC patients compared to advanced cases. TNF-α serum level was significantly higher in CRC patients than in controls (36.1 ± 8.4 pg/mL vs. 18.66 ± 11 pg/mL; *p* = 0.0000001). In the subgroup analysis by tumour–node–metastasis stages, the highest TNF-α level was found in stage IV (42.7 ± 12.5 pg/mL) and was significantly elevated compared to earlier stages of CRC and controls. The survival rate of CRC patients with low TNF-α serum level, estimated as median survival, was significantly higher than that of patients with high levels of TNF-α (38.4 vs. 7.761 months; log rank test *p* = 0.00015) In conclusion, we can affirm that TNF-α affects tumour development along with disease progression which has an impact on the survival of CRC.

## Introduction

Colorectal cancer (CRC) is one of the most common malignancies and represents a significant cause of morbidity and mortality worldwide. It is well known that the inflammatory process together with the genetic background has a great impact on the carcinogenesis.

Pro-inflammatory and anti-inflammatory cytokines play a key role in the regulation of inflammation. Tumour necrosis factor-alpha (TNF-α) is a pro-inflammatory cytokine, primarily released by activated monocytes/macrophages as well as tumour cells. TNF-α has several actions, such as stimulating the acute phase response, mediates the early inflammatory response and regulates the production of other cytokines, hemokines and endothelial adhesion molecules.[1,2] TNF-α also increases vascular permeability, leading to recruitment of activated leukocytes to the site of infection or injury, enhances oncogene activation, tumour cell invasion and angiogenesis.[3,4] TNF-α expression by tumour cells may be an efficient immunological escape mechanism by induction of apoptosis in immune cells resulting in a downregulation of the tumoural immune response. Despite the name and history of this cytokine, these properties make TNF-α a promoter of inflammation, angiogenesis and tumour dissemination; it could be considered as a tumour-promoting factor.[5]

Recent experimental and clinical studies on the role of TNF-α have demonstrated that the TNF-α is a key player in progression of human CRC.[6–9] Balkwill et al. have shown that if the level of TNF-α is elevated in patients with CRC they have a poor prognosis.[6] Levels of plasma cytokines, including TNF-α, have been shown to predict clinical outcome in patients with advanced CRC.[9]

In addition, some studies have reported that a substitution G/A at –308 position of TNF-α gene promoter is correlated with cytokine production and increased cancer risks. A-allele was associated with higher circulating TNF-α level and may alter the immune response.[10] Such gene polymorphisms that modify the cytokine level may contribute to cancer development and progression. Recent meta-analysis demonstrated that TNF-α –308G/A is a risk factor for developing cervical cancer [11] and breast cancer [12] in contrast to CRC.[13] Some studies demonstrated the lack of association between –308G/A polymorphism in TNF-α and risk of CRC.[14–16] However, to the best of our knowledge, the distribution of –308G/A polymorphism in TNF-α among Bulgarian CRC patients was not explored previously.

In respect to the above, the aim of the current case-control study was to investigate the effect of –308G/A polymorphism in TNF-α gene promoter on serum level of TNF-α and its correlation with cancer progression and survival in CRC patients in ethnic Bulgarian population.

## Materials and methods

### Subjects

A group of 119 Bulgarian patients with CRC were included in the study of distribution of the –308G/A TNF-α polymorphisms. Cases with new diagnosis of CRC attending the University hospital in Stara Zagora and an Oncology centre in Pleven, Bulgaria were selected. All patients had no previous diagnosis of inflammatory bowel disease or any of the known hereditary cancer syndromes. Patients did not receive chemotherapy or radiation therapy before surgery. The histopathological examination confirmed the diagnosis of cancer. Tumour grading and staging was performed according to the tumour–node– metastasis (TNM) classification. The patients’ group was composed of 71 (60%) male and 48 (40%) female. Different stages of CRC were found in both sex groups in approximately equal proportion (χ^2^ = 2.698, df = 3, *p* = 0.44). The mean age at diagnosis of male vs. female among the cases was 65.07 ± 10 years vs. 66.16 ± 11 years (*p* = 0.57). Demographic data and disease status of 119 CRC patients are presented in Table 1. The 177 age- and sex-matched no-CRC controls without any previous cancer diagnosis were selected from the same cohort as the corresponding cases. Patients from CRC and control groups with concomitant diseases (e.g. infectious diseases, inflammatory bowel disease, autoimmune conditions, etc.) known to affect cytokine expression and serum levels were excluded from the study.

**Table 1.** Demographic data and disease status of CRC patients at time of diagnosis.

	Stage I	Stage II	Stage III	Stage IV	Total
Number of patients (%)	14 (12%)	44 (37%)	43 (36%)	18 (15%)	119 (100%)
Male (%)	7 (10%)	28 (39%)	23 (32%)	13 (18%)	71 (60%)
Female (%)	7 (15%)	16 (33%)	20 (42%)	5 (10%)	35 (40%)
Age (mean ± SD) years	62.4±15	64.8±10.4	67.3±9.9	65.3±7.7	65.5±10.5

Blood specimens from the patients before surgery and no-CRC controls were collected in tripotassium EDTA sterile tubes for DNA isolation. For quantitative determination of TNF-α, serum samples were separated from venous blood at room temperature and stored at –70 °C until use.

Informed consent was obtained from all subjects and authorization was given by the Ethics Review Board of the Faculty of Medicine, Trakia University, Stara Zagora and Medical University, Pleven.

### Cytokine determination

The quantity determination of TNF-α in sera was performed by enzyme-linked immunosorbent assay (ELISA) kits (Invitrogen BioSource, Austria), following the manufacturer's protocol. Developed colour reaction was measured as OD units at 450 nm. The concentration of TNF-α was determined by using the standard curve constructed with the kit's standards and was expressed in pg/mL. The minimum detectable concentration of the TNF-α ELISA kit was less than 2 pg/mL.

### DNA extraction and genotyping of –308G/A polymorphisms in TNF-α gene

Genotyping for the –308G/A polymorphisms in TNF-α (rs1800629) was performed by restriction fragment length polymorphisms (RFLP)-PCR assay after amplification of 150 bp fragment with forward primer: 5′–AGG CAA TAG GTT TTG AGG GCC AT-3′ and reverse primer: 5′–TTG GGG ACA CAC AAG CAT CAA GG–3′. The cycling parameters were as follows: initial incubation step of 2 min at 95 °C; 35 cycles: 45 sec at 95 °C, 45 sec at 65 °C and 45 sec at 72 °C and a final extension step of 5 min at 72 °C completed the reaction. Amplified products were digested using 10 units of NcoI (Thermo Scientific) per reaction for 16h at 37 °C. The –308G allele yields two fragments, 128 bp and 22 bp, respectively. PCR amplification was performed in a GeneAmp PCR System 9700 (Applied Biosystems). Primers and reagents for PCR reactions were supplied by Metabion GmbH (Germany) and Thermo Scientific, respectively. PCR products and restriction fragments were visualized on an agarose gel stained with ethidium bromide (0.5 mg/mL). In each PCR run, heterozygous control template was used to ensure accuracy. For quality control, 10% of random selected samples containing both cases and controls were analysed a second time without finding any discrepancies.

### Statistical analysis

Statistical analysis was carried out using SPSS software, version 21 (IBM, Chicago, IL). The independence of qualitative outcomes was tested using Pearson's chi-square test and Fisher's exact test, where appropriate. Differences in serum levels of TNF-α between groups and across genotypes were assayed by one-way ANOVA, and if significant, Fisher LSD test for post-hoc pairwise comparisons was applied. The survival curves were made using the Kaplan–Meier method and comparison was with the log rank test. In all cases *p*-value less than 0.05 (two-tailed) was considered significant.

## Results

### Distribution of –308G/A polymorphism of TNF-α gene

Genotype and allele frequencies of –308G/A polymorphism in promoter of TNF-α gene were determined in 177 healthy donors and in 119 CRC patients from Bulgaria (Table 2). The genotype distribution for the investigated polymorphism was in agreement with Hardy­–Weinberg equilibrium in both groups (controls: *p* = 0.612 and cases *p* = 0.668).

**Table 2.** Genotype and allelic frequencies of –308G/A TNF-α polymorphism among CRC patients and healthy donors.

	CRC (*n* = 119)	Healthy donors (*n* = 177)	OR	95% CI	*p*
Allelic frequency					
−308G / −308А	0.857 / 0.143	0.876 / 0.124	1.174	0.71–1.95	0.513
Genotype distribution					
GG	88 (0.74)	135 (0.763)	1	Ref.	
AG	28 (0.235)	40 (0.226)	1.074	0.595–1.934	0.800
AA	3 (0.025)	2 (0.011)	2.301	0.305–20.126	0.354

The observed genotype frequencies of –308G/A TNF-α promoter polymorphism were similar among CRC patients and no-CRC controls (*p* = 0.64; df = 2). However, there was a trend of higher frequency of AA genotype (2.5%) among cases compared to controls (1.1%) and to allele-A (14.3% vs. 12.4%) but without statistical significance.

Furthermore, we investigated the association of –308G/A TNF-α polymorphisms and severity of CRC. The results are presented in Table 3. After grading and staging the cases according to TNM classification, homozygous genotype of variant allele-AA was more frequent in patients with early CRC (3.4%) compared to advanced CRC patients (2%) with OR = 2.19; 95% CI: 0.15–63.51; *p* = 0.519). As a result, allele-A was more frequent in early CRC patients compared to advanced cases (0.155 vs. 0.131; OR = 1.217; 95% CI: 0.56–2.67; *p* = 0.597). However, no statistically significant differences in distribution of –308G/A TNF-α polymorphism between different tumour stages were detected.

**Table 3.** Genotype and allelic frequencies of –308G/A TNF-α polymorphism among patients with different stages of CRC.

–308G/A polymorphism in TNF-α gene					
	GG	GA	AA	Allele-G	Allele-A
CRC stage	*n* (%)	*n* (%)	*n* (%)	*n* (%)	*n* (%)
Stage I (*n* = 14)	9 (64%)	5 (36%)	–	23 (82%)	5 (18%)
Stage II (*n* = 44)	33 (75%)	9 (20.5%)	2 (4.5%)	75 (85%)	13 (15%)
Stage III (*n* = 43)	34 (79%)	9 (54%)	–	77 (89.5%)	9 (10.5%)
Stage IV (*n* = 18)	12 (67%)	5 (28%)	1 (5%)	29 (81%)	7 (19%)
Early CRC – Stages I and II (*n* = 58)	42 (72.4%)	14 (24.2%)	2 (3.4%)	98 (84.5%)	18 (15.5%)
Advanced CRC – Stages III and IV (*n* = 61)	46 (75%)	14 (23%)	1 (2%)	106 (87%)	16 (13%)

In respect to limited number of homozygous AA genotype, statistical analyses were performed between individuals carrying the variant allele-A (genotype AG and AA) and individuals with homozygous GG genotype. However, no significant associations were observed between investigated polymorphism in TNF-α gene and CRC.

### Serum level of TNF-α

**Figure 1.**
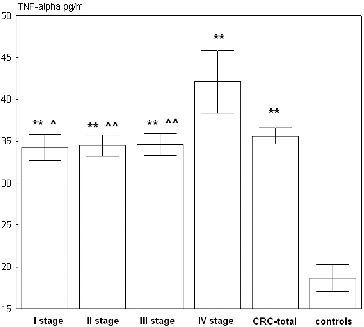
Serum levels of TNF-α among patients with different stages of CRC and unaffected controls. Note: The results are presented as mean value ± SE. ** *p* < 0.01, CRC patients vs. unaffected controls; ^ *p* < 0.05; ^^ *p* < 0.01 – CRC patients with different stages vs. stage IV of CRC

TNF-α serum levels were measured in 68 CRC patients and 47 controls by the ELISA method and are presented in Figure 1. Serum TNF-α levels in total group of CRC patients (36.1 ± 8.4 pg/mL) were significantly higher than those in the control group (18.7 ± 11 pg/mL, *p* < 0.0000001). After grading and staging the cases according to the TNM classification, the highest TNF-α level was found in stage IV of CRC (42.7 ± 12.5 pg/mL) and it was significantly higher when compared to the earlier stages of CRC and control group (*p* < 0.05). Serum TNF-α was approximately equal among stage I (34 ± 4 pg/mL); stage II (34.5 ± 6.3 pg/mL) and stage III (34.6 ± 6.7 pg/mL) of CRC and significantly elevated compared to the controls.

**Figure 2.**
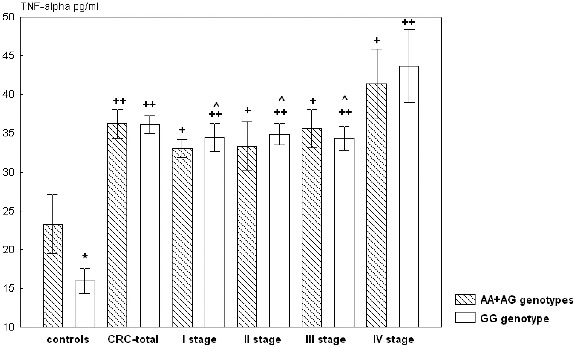
Serum levels of TNF-α among patients with different stages of CRC and unaffected controls in relation to genotypes of –308G/A polymorphism in TNF-α gene. Note: The results are presented as mean value ± SE. * *p* < 0.05 – unaffected controls with GG genotype vs. AG + GG genotype; ^ *p* < 0.05 – CRC patients with different stages vs. stage IV of CRC with the same genotype; + *p* < 0.05; ++ *p* < 0.01 – CRC patients vs. unaffected controls with the same genotype

Moreover, we investigated the relationship of serum TNF-α and genotypes of –308G/A polymorphisms in TNF-α gene in CRC patients and healthy donors (Figure 2). In respect to limited number of homozygous AA genotype, we compared TNF-α level between individuals carrying the variant allele-A (genotype AG and AA) and individuals with homozygous GG genotype in cases and controls. No association was observed between carrying the variant allele-A of –308G/A polymorphism and serum TNF-α in total group of CRC patients. Additionally, no significant difference in TNF-α level was observed between the patients with different genotypes of –308G/A polymorphism within the same stage of CRC. The highest level of TNF-α was observed in the stage IV of CRC with genotype-GG (43.7 ± 14 pg/mL). CRC patients in stage IV of CRC with GG genotype showed significantly elevated levels of TNF-α compared to all earlier stages of CRC with the same genotype (for stage I: 34.4 ± 4.8 pg/mL, *p* = 0.042; for the stage II: 34.9 ± 6 pg/mL, *p* = 0.018; for stage III: 34.4 ± 7 pg/mL, *p* = 0.013). The same tendency was observed for AA + AG genotypes without reaching statistical significance.

However, among control group, the carrying of variant allele-A was associated with increased serum level of TNF-α. Significant elevated serum TNF-α was detected in individuals with genotypes-AA + AG (23.3 ± 12.1 pg/mL) compared to GG genotype (15.9 ± 9.6 pg/mL; *p* = 0.024).

### Survival of CRC patients stratified by TNF-α serum concentration and genotype of –308G/A polymorphism in TNF-α gene

**Figure 3.**
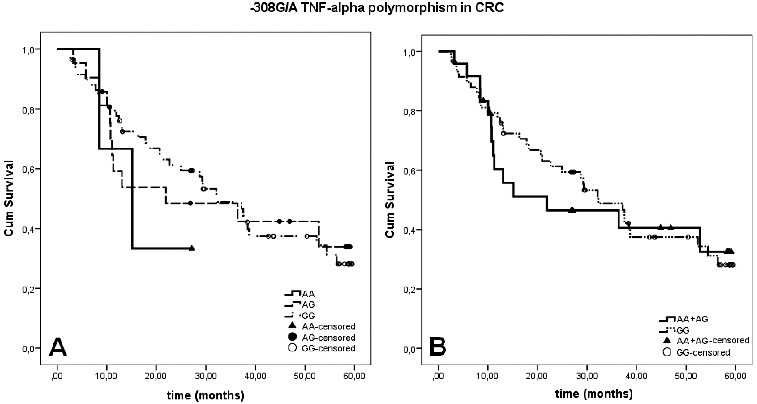
The Kaplan–Meier survival curves of CRC patients stratified by genotype of –308G/A polymorphism in TNF-α gene. Panel A presents the three genotypes separately, and paned B presents the homozygous of wild allele GG genotype and genotypes carrying at least one of the variant alleles (AA + AG).

The Kaplan–Meier survival curves representing the survival rates of the patients according to their genotypes are presented in Figure 3. Different genotypes of the –308G/A polymorphism in TNF-α had no significant impact on the overall survival-rate of CRC patients (χ2 = 0.621; df = 2, log rank test *p* = 0.733). However, it should be mentioned that a poor survival rate among CRC patients with genotype-AA (33.3%) was observed when compared to patients with AG and GG genotypes, without reaching statistical significance. Additionally, no significant difference was observed between CRC patients with GG genotype and those with genotypes carriers of variant allele-A (AA + AG) with χ2 = 0.061; df = 1, log rank test *p* = 0.805. Stratification by tumour stage according to TNM classification showed no significant association between investigated polymorphism and survival-rate of CRC patients.

**Figure 4.**
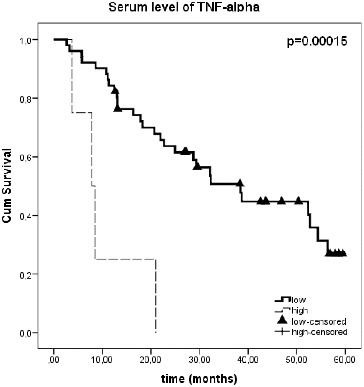
The Kaplan–Meier survival curves of CRC patients according to serum level of TNF-α. Serum TNF-α level of less than 51.66 pg/ml was classified as low, and levels more than 51.66 pg/ml was classified as high. Note: The threshold level was calculated as mean + 3*SD of serum concentration of TNF-α among control group.

We further evaluated the correlation of serum level of TNF-α and survival in CRC patients. Taking a 51.66 pg/mL, mean value + 3* standard deviation of serum level detected among control group, as the threshold level, the total group of CRC was subdivided to patients with low TNF-α level (below 51.66 pg/mL) and patients with high level (above 51.66 pg/mL). The pre-operative serum TNF-α concentration was high in four patients. Three of patients with high TNF-α were in stage IV of CRC and one was in stage III of CRC. Interestingly, all four patients with high serum TNF-α died during the follow-up period of the present study. As a result, the survival rate of CRC patients with high TNF-α serum level was significantly lower than that of patients with low levels of TNF-α. CRC patients with high TNF-α concentration showed a poor survival of 0% as compared to 43.1% with low TNF-α concentration (log-rank test *p* = 0.00015; Figure 4). For patients with low TNF-α serum level, the estimated median survival was 38.4 months (95% CI: 26.3–50.6) and was significantly higher than those for patients with high levels of TNF-α (7.761 months; 95% CI: 3.120–12.401).

## Discussion

In the present study, we explored the role of serum TNF-α and polymorphism –308G/A in TNF-α gene in development, progression and survival of CRC among Bulgarian population.

We found that the investigated polymorphism is not significantly associated with genetic predisposition of CRC risk. This is in agreement with other previous studies demonstrating the lack of association between polymorphism –308G/A in TNF-α gene and CRC risk.[14–16] The distribution of different TNF-α alleles in our entire CRC population did not differ from the distribution in other European population.[13] According to our knowledge, there was no other report in the literature pointing on this polymorphism in Bulgarian CRC patients. Therefore, we have compared the observed frequencies among control group: GG-76%; AG-23%; AA-1% with those determined by other authors among different control group of 77 unrelated healthy individuals from Bulgaria GG-78%; AG-21%; AA-1%.[17] Based on these similarities, we can conclude that ethnic Bulgarians do not differ from other Caucasians in frequency of –308G/A genotypes. However, there was a trend of higher frequency of AA genotype among cases compared to controls (2.5% vs. 1.1%) and to allele-A (14.3% vs. 12.4%) but without statistical significance. Furthermore, we demonstrated that TNF-α –308G/A polymorphism might be associated with progression of CRC. The frequencies of AA genotype were elevated in patients with early compared to advanced CRC patients.

In addition, we examined the effect of –308G/A TNF-α polymorphism on survival rate of CRC patients. A tendency of poor survival rate was seen in patients with homozygous genotype-AA compared to patients with heterozygous and homozygous – GG genotypes, although the statistical significance was not reached. A similar trend was also observed for genotypes carrying the variant allele (AA + AG) compared to GG genotype. Obviously carrying AA genotype has a greater disadvantage in respect to survival among CRC patients than the carrying of variant allele-A in heterozygous genotype.

As it is well known, when a selectively favourable gene substitution occurs in a population, changes in variant allele frequencies will affirm depending on the aggregate functional effect of the substitution. Bearing in mind the above simultaneously with the extremely low frequency of variant homozygotes in our current study, as well as in previous studies among other European populations,[13] we could hypothesize that the polymorphism –308G/A in TNF-α gene is still in process of evolutionary maintenance in human populations. Therefore, we must emphasize that in the CRC patients group the frequency of genotype-AA was twofold more frequent (2.5%) compared to that in the control group (1.1%), although the absolute number is small and no statistical significance was observed. Further investigations are needed to reveal the accurate relationship, if any, between the CRC progression and TNF-α –308G/A polymorphism. Although –308G/A polymorphism in TNF-α was not significantly associated with risk, progression and survival-rate of CRC in our present case-control study, we established its functional effect on cytokine serum level. We observed a significantly high TNF-α level in genotypes carrying at least one variant allele (AA + AG) compared to GG genotype in the control group. This result is in agreement with a previous study, which demonstrated greater transcriptional activity of –308A allele of the TNF-α gene.[10] However, in contrast to control group, in CRC patients’ serum TNF-α level were similar across different genotypes. An explanation of this result could be that elevated TNF-α in process of colon carcinogenesis due to many other inducers of TNF-α synthesis, evoke activation of different transcription factors which mask the individual effect of –308G/A polymorphism in TNF-α.

## Conclusion

The strong evidence of the TNF-α role in CRC development is the fact that an approximately twofold increase of serum TNF-α in CRC patients was found when compared to controls, regardless of TNF-α –308G/A polymorphism. In addition, the highest level of TNF-α was found in stage IV of CRC. Evaluating the relationship between the survival and serum TNF-α, it was observed that patients with high level (above 51.66 pg/mL) showed significantly inferior survival capability than patients with lower TNF-α levels (log rang, *p* = 0.00015). Our results are in agreement with other previous studies.[6–9,18] Kim et al. demonstrated that circulating TNF-α is associated with increased risk of colorectal adenoma.[18] A study by Grimm et al. indicated that TNF-α expression in CRC specimen and the CRC cell line HT-29 is associated with cancer progression and reduced tumour specific survival among patients with high TNF-α expression compared to patients with low TNF-α expression.[8] Our present study demonstrated elevated serum level in all CRC patients with the highest level in stage IV of CRC associated with poor prognosis of CRC survival. We could assume that endogen TNF-α plays a negative role in CRC patients, favouring the growth of CRC and tumour progression and it should therefore be considered as a tumour-promoting factor rather than cytokine with anti-tumoural activities.

We could conclude that TNF-α has a great impact on development, progression and survival of CRC. The elevation of serum TNF-α level was associated with advanced stage of CRC and reduced survival of CRC patients.
